# Effective policy initiatives to constrain lipid-lowering drug expenditure growth in South Korea

**DOI:** 10.1186/1472-6963-14-100

**Published:** 2014-03-03

**Authors:** Green Bae, Chanmi Park, Hyejin Lee, Euna Han, Dong-Sook Kim, Sunmee Jang

**Affiliations:** 1 Review and Assessment Research Institute, Health Insurance Review and Assessment Services, 267 (Seocho-dong) Hyoyeong-ro, Seocho-gu, Seoul 137-706, South Korea; 2 Bioethics Policy Studies, Ewha Womans University, 314 Posco, 52, Ewhayeodae-gil, Seodaemun-gu, Seoul 120-750, South Korea; 3 College of Pharmacy, Yonsei University, 162-1 Songdo-dong, Yeonsu-gu, Incheon 406-840, South Korea; 4 College of Pharmacy & Gachon Institute of Pharmaceutical Sciences, Gachon University, 191 Hambakmoe-ro, Yeonsu-gu, Incheon 406-799, South Korea

## Abstract

**Background:**

The rapid growth of prescription drug expenditures is a major problem in South Korea. Accordingly, the South Korean government introduced a positive listing system in 2006. They also adopted various price reduction policies. Nevertheless, the total expenditure for lipid-lowering drugs have steadily increased throughout South Korea. The present study explores the factors that have influenced the increased expenditures of lipid-lowering drugs with a particular focus on the effects of statins in this process.

**Methods:**

This paper investigates the National Health Insurance claims data for prescribed lipid-lowering drugs collected between January 1, 2005 and December 31, 2009. We specifically focused on statins and assessed the yearly variation of statin expenditure by calculating the increased rate of paired pharmaceutical expenditures over a 2 year period. Our study classified statins into three categories: new entrants, core medicines and exiting medicines. For core medicines, we further examined influencing factors such as price, amount of drugs consumed by volume, and prescription changes (substitutes for other drug).

**Results:**

Statin expenditure showed an average annual increase of 25.7% between 2005 and 2009. Among the different statins, the expenditure of atorvastatin showed a 36.6% annual increase rate, which was the most dramatic among all statins. Also we divided expenditure for core medicines by the price factor, volume factor, and prescription change. The result showed that annual weighted average prices of individual drug decreased each year, which clearly showed that price influenced statin expenditure in a negative direction. The use of generic drugs containing the same active ingredient as name-brand drugs increased and negatively affected statin expenditure (Generic Mix effect). However, the use of relatively expensive ingredients within statin increase, Ingredient Mix effect contributed to increased statin expenditure (Ingredient Mix effect). In particular, the volume effect was found to be critical for increasing statin expenditure as the amount of statin consumed increased steadily throughout the study period.

**Conclusions:**

The recent rapid increase in statin expenditure can largely be attributed to an increase in consumption volume. In order to check drug expenditures effectively in our current situation, in which chronic diseases remain steadily on the rise, it is necessary to not only have supply-side initiatives such as price reduction, but also demand-side initiatives that could control drug consumption volume, for example: educational programs for rational prescription, generic drug promotional policies, and policies providing prescription targets.

## Background

In Korea, the National Health Insurance prescription drug expenditure has increased about 13.2% annually for the past decade (2001–2010) [1]. This cost makes it a major threat to the sustainability of Korea's National Health Insurance program. Accordingly, the Korean government has introduced various policies to check these costs that have continued to grow since 2006. In December 2006, they introduced the positive listing system, inducing pharmaceutical companies to consider cost-effectiveness as one of their major concerns [2], and required them to cut the price of original drugs by 20% when their patents expire. Also, from 2007 to 2010, they re-evaluated the cost-effectiveness of already listed drugs and lowered the cost of drugs with low cost-effectiveness or delisted them [3]. This re-evaluation of listed drugs was done mainly to drugs for chronic diseases like hyperlipidemia and hypertension. In 2009, pharmaceutical companies also introduced the price-volume agreement system.

If we divide factors for prescription drug expenditure into two categories – the price factor and the volume factor – most pharmaceutical regulations introduced in Korea between 2006 and 2009 utilized supply-side initiatives like the price and the drug-listing decision method [4]. As a result, the government rarely applied demand-side initiatives, which efficiently control prescription drug expenditures by checking their volume on the stage of drug prescription and usage. In this case, if the main reason for the increase in prescription drug expenditure wasn't price, but volume, then, the effect of policies focusing on price-cuts and unaccompanied by demand-side initiatives would remain limited and stay short-term in nature. Therefore, in order to approach the issue of constraining prescription drug expenditure increases, we need first to examine the various factors surrounding the phenomenon, including price, volume, and prescription changes, and their influence on prescription drug expenditure increases [5,6].

When we examine the influences the three above-mentioned factors have on changes in prescription drug expenditures, we find that their influence varies according to the drug. This study examines the influence lipid-lowering drugs have on the prescription drug expenditure increase. The lipid-lowering drug expenditure increased sharply, doubling from 2005 to 2009 [7]. This drug was also influenced the most by various policy changes during this period. As the patent expired in August 2008, the price of the original atorvastatin lowered by 20%. In particular, as a result of re-evaluating the listed drugs, which indicated that there were no significant differences among statins in their effects on lowering LDL, the prices of all statins above a certain price were lowered two times by 5% ~ 37.5% (an average 10.3%) in April 2009 and in January 2010 [4]. Despite these changes, lipid-lowering drug expenditures continued to grow steadily [7]. It is necessary for us first to look into various factors influencing the lipid-lowering drug expenditure increase in detail. Results of the current study could offer useful suggestions for future plans to more effectively constrain the lipid-lowering drug expenditure increases.

## Methods

### Data

For our study we used the Korean National Health Insurance Claims Database (KNHICD). The Korean National Health Insurance plan provides coverage for 97% of the entire South Korean population. NHI benefits not only cover outpatient, inpatient and emergency care, but also prescription drugs dispensed at pharmacies. We focused on lipid-lowering drugs that were prescribed and dispensed in outpatient settings between 2005 and 2009.

We defined lipid-lowering drugs in this study as drugs with an Anatomical-Therapeutic Classification System (ATC) code that corresponded to ‘C10 LIPID MODIFYING AGENTS’. Given that statins represent more than 90% of all pharmaceutical expenditures for lipid-lowering drugs, we decided to focus on statins alone as our final study target. We estimated the price of an individual drug using an annual weighted average price calculated as the annual expenditure divided by annual consumption. The amount of statin consumed was computed based on the Defined Daily Dose of the World Health Organization (WHO-DDD) at 2010 [8-10]. With the sole unit of DDD, it was feasible to compare the amount of drug consumed among identical active ingredients even when they had different strength and administration routes.

The current study was approved of by the Institutional Review Board (IRB) of Health Insurance Review and Assessment Services (HIRA).

## Method

**Figure 1:**
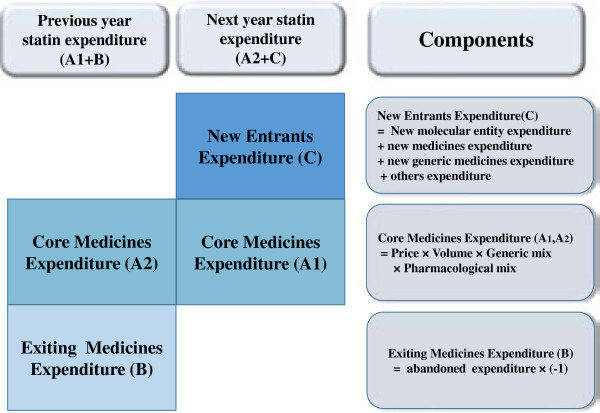
Framework of the statin expenditure analysis.

We calculated the yearly variation of statin expenditures by measuring the increased rate of paired pharmaceutical expenditures for a 2 year period. We added "Expenditure" only to the price of the drugs and did not include compounding fees, etc. In Korea, patient co-payment is a certain percentage of total expenditures that compose drug prices plus compounding fees. Thus, "expenditures" in this study were defined as total drug cost including some of the patient co-payment. We adjusted for inflation by dividing the monetary time series by the Consumer Price Index (CPI) with the CPI being scaled so that 2009 value was 1.0 [11]. And we used exchange rate listed by the Bank of Korea (Additional file 1) [12]. Referring to the study of Chernew (2001), we categorized statins as new entrants, core medicines or exiting medicines (Figure 1) [6]. We defined new entrants as medicines that were not used during the previous year but were used in the base year. Core medicines were drugs that were used during both years. Exiting medicines were defined as drugs that were used during the previous year but were not used in the base year. We further divided new entrants into new molecular entities, new medicines, new generic medicines, and others. New molecular entities referred to compounds that the NHI covered during the specified year. We defined new medicines as drugs recently listed with new administration routes or chemical compositions but with their active ingredients already included in the NHI benefit list for the specified year. New generic medicines were primarily identified as newly launched generic drugs for which the original patent had expired. The rest of the drugs within the new entrants were classified as "others". These medications were already listed in NHI benefit list but were not used during the comparable period.

The number of active ingredients in our study was 19(183 drugs) in 2005 but this was expanded to 30 active ingredients (271 drugs) in 2009 (Table 1).

**Table 1:** Number of drugs and active ingredients among statins*

	**Year**	**2005**	**2006**	**2007**	**2008**	**2009**
Number of drugs	Total	183	207	217	227	271
	**Core medicines (A)**		167	192	176	204
	**Exiting medicines (B)**		16	15	41	23
	**New entrants (C)**		40	25	51	67
	- New molecular entities		0	0	0	1
	- New medicines		1	0	1	5
	- New generic medicines		9	13	37	29
	- Others		30	12	13	32
Number of active ingredients	Total	19	22	23	24	30
	**Core medicines (A)**		19	22	23	24
	**Exiting medicines (B)**		6	4	8	6
	**New entrants (C)**		13	8	12	17
	- New molecular entities		0	0	0	1
	- New medicines		1	0	1	5
	- New generic medicines		6	5	6	5
	- Others		6	3	5	6

*This table includes only those statins that were prescribed by doctors out of statins on the Korean National Health Insurance benefit list.

Table 2 divided statins on the KNHI (Korea National Health Insurance) benefit list into 7 different types according to their ingredients, additionally subdividing them into original and generic drugs. These included all statins on the KNHI benefit list between 2005 and 2009, regardless of whether they were actually prescribed or not. This table indicates that many atorvastatin generic drugs were placed on the benefit list at around same time in July 2008 when the patent expired.

**Table 2:** Number of statins on KNHI benefit list (2005–2009 year)*

**Year**	**2005**		**2006**		**2007**		**2008**		**2009**	
**Statins**	**Original**	Generic**	**Original**	Generic**	**Original**	Generic**	**Original**	Generic**	**Original**	Generic**
Simvastatin (10, 20, 40, 80 mg)	3	125	3	134	3	135	3	118	4	100
Lovastatin (20 mg)	1	54	1	56	1	52	1	35	1	25
Pravastatin (5,10,20,40 mg)	0	49	0	50	0	81	0	56	0	48
Fluvastatin (20,40,80 mg)	3	0	3	0	3	0	3	0	3	0
Atorvastatin (10,20,40,80 mg)	3	0	3	0	3	0	4	42	4	54
Rosuvastatin (5,10,20 mg)	2	0	3	0	3	0	3	0	3	0
Pitavastatin (2 mg)	1	0	1	0	1	0	1	0	1	0
Total	241		254		282		266		243	

*They include all statins on the KNHI benefit list between 2005 and 2009, regardless of whether they were actually prescribed or not.

**The original and generic drugs were categorized only according to their ingredients and contents. Different administration routes, dosage forms, and strength of unit were not taken into account.

We computed the yearly variation of statin expenditures starting in 2005 by calculating the increased rate of paired pharmaceutical expenditures observed over a two-year period. One can express spending increases for statins between the previous year (0) and base year (1) with the following equation when the targeted drugs were classified as new entrants, core medicines, and existing medicines:

$$\frac{S^{1}}{S^{0}}-1=\theta^{C}\times \left(\frac{C^{1}}{C^{0}}-1\right)+\theta^{A}\times \left(-1\right)+\frac{N^{1}}{S^{0}}$$

$S^{1},S^{{}^{0}}$: Total drug expenditures for the base year (1) and previous year (0)

*C*^1^, *C*^0^: Core medicines expenditures for the base year (1) and previous year (0)

*N*^1^: New entrants expenditures for the base year (1)

*A*^0^: Exiting medicines expenditures for the previous year (0)

$\theta^{C}:\frac{C^{0}}{S^{0}}$, Expenditure proportion for core medicines in previous year

$\theta^{A}:\frac{A^{0}}{S^{0}}$, Expenditure proportion for exiting medicines in previous year

$\frac{N^{1}}{S^{0}}$: Increased rate for new entrants

We further divided expenditures for core medicines by the price of medication, amount of drugs consumed by volume, and prescription changes, which we have illustrated in the equation slightly further below. There are two possible factors that could have led to prescription changes: Generic Mix or Ingredient Mix. Generic Mix refers to cases where prescribed drugs were substituted for other medicines with identical generic names. Since the two drugs shared the same generic name, their ingredients, dosage type and action mechanisms were identical even as their prices differed. We determined whether pharmaceutical expenditures for 2 years showed any changes in the proportion of high/low priced drugs with the same ingredients. Ingredient Mix refers to the cases in which the existing drugs were replaced with other drugs in the same therapeutic category. For example, if a patient changed his or her drug from simvastatin to atorvastatin, one could express this as an Ingredient Mix since both medications belonged to the statin classification.

$$\begin{array}{l} \text{Core}\;\text{medicine}\;\text{expenditure}\;\left(C\right)\\ \;=\Sigma (\text{individual}\;\text{drug}\;\text{price}\times \text{generic}\;\text{mix}\\ \;\times \text{Ingredient}\;\text{mix}\times \text{total}\;\text{volume}) \end{array}$$

$$C=\sum_{i=1}^{m}\sum_{j=1}^{n_{i}}\left(p_{\mathrm{ij}}\times \frac{q_{\mathrm{ij}}}{\sum_{j}^{n_{i}}q_{\mathrm{ij}}}\times \frac{\sum_{j}^{n_{i}}q_{\mathrm{ij}}}{\sum_{i}^{m}\sum_{j}^{n_{i}}q_{\mathrm{ij}}}\times \sum_{i}^{m}\sum_{j}^{n_{i}}q_{\mathrm{ij}}\right)$$

*p*_*ij*_: price of *j* th drugs within *i* th component

$\frac{q_{\mathrm{ij}}}{\sum_{j}^{n_{i}}q_{\mathrm{ij}}}$: share of *j* th drugs within *i* th component

$\frac{\sum_{j}^{n_{i}}q_{\mathrm{ij}}}{\sum_{i}^{m}\sum_{j}^{n_{i}}q_{\mathrm{ij}}}$: share of *i* th component

$\sum_{i}^{m}\sum_{j}^{n_{i}}q_{\mathrm{ij}}$: total dose of all drugs.

## Results

Our study demonstrated that statins accounted for 90% of total lipid-lowering drug expenditures. Annual expenditures for statins increased from 193 million won (188 million USD) in 2005 to 483 million won (378 million USD) in 2009 (an annual average of 25.7% during the study period). The annual statin DDD also showed an increase of 36.4% (from 129 million DDD in 2005 to 445 million DDD in 2009) while statin expenditure per DDD decreased 7.9% (1,506 won (1.47 USD) in 2005 to 1,085 won (0.85 USD) in 2009) (Table 3). As the patent expired in August 2008, the price of the original atorvastatin lowered by 20%. Also, as a result of re-evaluating the listed drugs, its insurance price lowered by an average of 11% in April 2009. Such a price reduction influenced the continuous decreasing of statin expenditure per DDD.

**Table 3:** Changes in annual DDD and drug expenditures (year-to-year rates of increase in parenthesis)

		**2005**	**2006**	**2007**	**2008**	**2009**	**2005-2009**	**Annual**
Statin	Sum of DDD	128,545,620	193,278,414 (50.36%)	274,288,699 (41.91%)	344,893,058 (25.74%)	445,426,185	246.51%	36.44%
	Drug expenditure per DDD (unit: won)	1,506	1,392	1,276	1,185	1,085	−27.95%	−7.87%
	Total statin expenditure (unit: million won)	193.6	269.0 (38.96%)	350.0 (30.12%)	408.8 (16.82%)	483.1 (18.16%)	149.57%	25.69%
All Lipid-lowering drugs	Sum of DDD	154,832,421	224,516,146 (45.01%)	314,095,889 (39.90%)	392,818,319 (25.06%)	506,311,055 (28.89%)	227.01%	34.47%
	Drug expenditure per DDD (unit: won)	1,349	1,285	1,206	1,136	1,052	−22.02%	−6.03%
	Total expenditure (unit: million won)	208.9	288.5 (38.09%)	378.7 (31.27%)	446.1 (17.82%)	532.5 (19.36%)	154.93%	26.36%

* The drug expenditure was adjusted for inflation.

**Figure 2:**
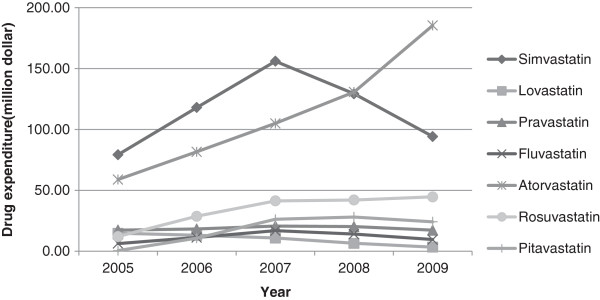
Expenditure trends for the seven different statins.

For a detailed analysis, we calculated drug expenditure percentages according to seven different statins. Our result showed that the percentage of simvastatin was the highest (41.9%) in 2005, followed by atorvastatin (31.2%) and pravastatin (9.2%). However, during the 4 year study period, simvastatin and lovastatin declined to 24.9% and 0.9% respectively. On the other hand, the percentage of atorvastatin increased rapidly to 49.0%, and the percentage of rosuvastatin rose to 11.8%. Atorvastatin particularly showed a rapid increase after 2008 when the original patent expired and generic drugs began to appear on the market. Pitavastatin use fluctuated slightly, increasing from 3.8% in 2006 to 7.6% in 2008, but decreasing to 6.4% in 2009 (Figure 2) (Additional file 2).

**Figure 3:**
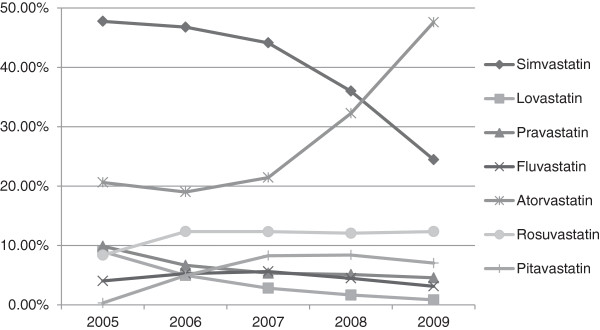
DDD proportion changes for each statin.

We also compared the amount of the seven statins consumed throughout this study. In 2005, simvastatin occupied 47.8% of the total statin DDD (Defined Daily Dose)followed by atorvastatin (20.6%), pravastatin (9.9%), lovastatin (9.0%), and rosuvastatin (8.4%). Our analysis demonstrated that there was significant fluctuation among the DDD proportion of the seven statins during the 4 year study period. The atorvastatin share, which ranked second in 2005, increased dramatically to 47.6% of the entire statin DDD in 2009. The percentage for simvastatin, on the other hand, plummeted to 24.5%. Use of rosuvastatin showed a slight increase (DDD proportion of 12.4%). In contrast, the DDD proportion of pravastatin decreased to 4.6% while lovastatin dropped to 0.9% (Figure 3).

Lastly, we explored the significant factors that influenced drug expenditure changes during the study period. We found that core medicines contributed the most to statin expenditure increases. Exiting medicines led the expenditure decrease of 13.4% in 2006 but had no significant influence thereafter. New entrants positively affected statin expenditure between 2006 and 2008 (from 2.7% to 10.5%) but rarely affected it in 2009 (1.0%).

Drug prices, which showed a continuous decrease, seemed to reduce the expenditures for statin. Especially, price factor contributed the most statin expenditure decreases, because insurance price of statins lowered by an average of 11.0% in 2009. However consumption volume increased annually and contributed to a substantial increase in statin expenditures. Since the patent for atorvastatin expired in 2008 and use of generic drugs increased, Generic Mix drove a statin expenditure decrease while the Ingredient Mix effect led to an expenditure increase of 5.3% (assuming the average rate of increase was 100%). This resulted from an increased use of more expensive active ingredients within the statin (Table 4).

**Table 4:** Effect of different factors on statin expenditure changes*

	**2005**	**2006**	**2007**	**2008**	**2009**	**Average**
Year-on-year increased statin expenditure rate (%)* (T)		**35.92**	**26.90**	**11.60**	**14.99**	**21.97**
**Core medicines (**year-to-year rates of increase: **%, A)**		**46.62**	**23.61**	**2.32**	**14.17**	**18.24**
- Price (△P)		−7.18	−0.33	−0.22	−6.21	−2.98
-Within-component mix (generic mix)		5.36	−2.12	−1.77	−2.06	−0.90
-Between-component mix		4.54	0.83	0.10	1.19	1.36
(Ingredient mix)						
- Volume (△Q)		47.94	32.21	5.04	21.58	23.48
**New entrants (**year-to-year rates of increase: **%, B)**		**2.69**	**3.30**	**10.54**	**0.96**	**6.43**
- New molecular entities		0.00	0.00	0.00	0.00	0.00
- New medicines		0.00	0.00	0.01	0.34	0.12
- New generic medicines		1.87	3.23	10.53	0.30	5.13
- Others		0.82	0.07	0.00	0.32	0.26
**Exiting medicines (**year-to-year rates of increase: **%, C)**		**−13.39**	**0.00**	**−1.26**	**−0.14**	**−2.70**

*The drug expenditure was adjusted for inflation.

## Discussion

Recently, hyperlipidemia has been on the rise in South Korea. Between 2005 and 2010, the prevalence of hyperlipidemia among people over 30 increased from 8.0% to 13.5%, while the prevalence of hypertension and diabetes rose from 24.6% to 26.9% and from 9.1% to 9.7% respectively [13]. Additionally, drug expenditures for lipid-lowering drugs doubled between 2005 and 2009. Moreover, compared to 2009, the use of lipid-lowering drugs in South Korea increased 27.4% in 2011 [7].

As is well known, hyperlipidemia is a major risk factor in contracting cardiovascular diseases. Hobbs (2004) reported that there is a more than 30% chance of cardiovascular disease development when cholesterol levels increase by 10%. If cholesterol levels increase by 30%, the risk of cardiovascular disease doubles [14]. In order to properly control hyperlipidemia, various recommendations have been made that include receiving lipid-lowering drugs along with maintaining a healthy diet and getting ample exercise [14-19].

Considering the notable effects of cardiovascular disease prevention, hyperlipidemia patients should take lipid-lowering drugs when necessary. In situations where the number of hyperlipidemia patients are rapidly increasing, the resulting increase in lipid-lowering drug prescriptions means an increasing burden to prescription drug costs. This study was designed to find a more effective way to constrain lipid-lowering prescription drug expenditures by examining the various primary factors influencing increases in expenditures.

By focusing on statins, which accounted for 90% of both lipid-lowering drug expenditures and consumption volume, we sought to identify the factors that drove expenditure increases. For our detailed analyses, we examined price, the amount of drugs consumed (volume), and prescription changes (substitutes for other drugs). For the amount of drugs consumed, we applied the DDD in order to standardize the volume of drugs with identical active ingredients regardless of their different administration routes, dosage forms, or strength and unit. Our current study's findings implied that increased expenditure for lipid-lowering drugs in South Korea was driven mainly by core medicines. Further analyses revealed that drug price consistently lowered statin expenditures while Generic Mix (the relative gap between consumption of name-brand and generic drugs) also reduced statin expenditures. This reflected the increasing use of generic drugs and could explain why most of the new entrants were considered ‘new products (generic).’ Additionally, prescription changes raised statin expenditures slightly due to an increased use of relatively expensive ingredients (Ingredient Mix effect). The results of this study also demonstrated that volume increases drove statin expenditures increases. Previous investigations analyzing South Korean drug expenditures from 2001 to 2006 also reported that volume increased due to an increasing number of patients, with prescriptions and prescribed days also acting as a main contributor [20-22]. Characteristically, after 2008, when the atorvastatin patent expired and various generic drugs began to appear on the market, atorvastatin expenditures increased significantly. This appears to be due to the fact that the production of generic drugs opened the possibility for doctors to prescribe a wider variety of lipid-lowering drugs. This conclusion is consistent with findings from a previous study by Kwon (2013), which found that the release of generic drugs on the market tended to not only replace the original drug market, but to also increase the overall market share for medication composed of essentially the same ingredients [4].

As mentioned in our introduction, the price of lipid-lowering drugs decreased during two significant periods between 2005 and 2009. Nevertheless, overall lipid-lowering drug expenditures continued to increase during 2008 and 2009. This was due to increases in the number of patients using these drugs and the number of their administration dates, as is shown in the increase in the entire DDD. In other words, the increase in lipid-lowering drug consumption was the main factor for expenditure increases. Therefore, it is clear that supply-side initiatives focusing on lowering prices would not be enough to effectively constrain drug expenditure increases.

Godman [23] argues that taking only one between supply-side initiative and demand-side initiative results in a limited effect on the constraint of prescription drug increases. Furthermore, one can maximize prescribing efficiency only when combining the two initiatives. He also argues that combining various demand-side initiatives amplifies the effect. In fact, the kind of increase in statin utilization witnessed in Korea is similar to statin utilization trends across Europe [23]. However, while statin use increased in the U.K. and Sweden, accompanying expenditures did not increase proportionally. One can impute these efficient expenditure numbers to their use of combined demand-side measures such as target prescription, compulsory INN prescription, positive and negative financial incentives, and mandatory generic substitutions. In Germany as well, in order to constrain the prescription drug expenditure and encourage generic drug prescription, authorities introduced target prescription as a cost-saving measure. This system was accompanied by offering immediate feedback so that doctors could monitor their own performance. It was through this combination of target prescription accompanied by educational methods and introducing a price-reference system that Germany was able to activate a relatively cheaper generic drug market [24,25]. In Korea, where the number of individuals with chronic illnesses have continued to rise, the authorities have made efforts to develop demand-side initiatives in addition to supply-side initiatives like lowering prices in order to effectively constrain prescription drug expenditure increases. For example, there is the prescribing incentive scheme (IPS), a system in which doctors are offered 30% of the saved drug cost if they voluntarily improve their prescription behavior and save drug costs. Also, in April 2012, Korea introduced a new price system where the highest price of both original and generic drugs is lowered to 53.55% of the original drug price, twelve months after the date the generic drug was listed and at the same time as the original drug’s patent expired. Because of this, the difference between the prices of original and generic drugs disappeared as well as the difference in co-payments. One could expect that the generic drug market could shrink slightly if one were to introduce this system. These initiatives clearly had some significant effect on limiting the prescription drug expenditures. Nevertheless, it would still be preferable to introduce more diverse demand-side initiatives to continue checking prescription drug expenditure increases and to maximize prescription efficiency.

The present study had several limitations. First, it was difficult to accurately measure drug usage volume. We confined our study to medications used for hyperlipidemia and measured consumption volume by adopting the DDD. However, drug consumption data presented in DDDs only gave a rough estimate of consumption and not an exact picture of actual use [26]. Second, the study method we used was not sufficient for assessing the impact of introducing new drugs on changes in drug expenditures [27]. Every year, pharmaceutical companies launch newly developed drugs and products while removing many existing drugs from the market. For this reason, studies are usually conducted either every year or once every 3 years, a short period for new entrants to acquire a certain level of the market share. Therefore, one might underestimate the influence of new entrants on current drug expenditures with those of the previous year. However, statins had few “new molecular entities” among “new drugs,” and most of the new drugs in our study sample were generic and had already acquired expired patents. Therefore, this common limitation that could have led to us underestimating our results would not have impacted our analysis.

## Conclusions

As chronic diseases including hyperlipidemia increase steadily in South Korea, it is necessary to introduce not only supply-side measures but also various demand-side measures such as educational courses for rational prescription, programs promoting cost-effective prescription drug usage, and a prescription target system to effectively constrain prescription drug expenditure increases.

## Competing interests

The authors declare that they have no competing interest.

## Authors’ contributions

GB (the first author) was a principal collaborator in designing this study. She undertook data analysis tasks of this study and the prepared the manuscript for submission. SJ (Corresponding author) was actively involved in this study's planning and enactment. She led the design of this research and contributed to the interpretation of the results. CP took part in designing the research and data analysis. She contributed to interpreting the results and the discussions. HL participated in analyzing the data and shared the interpretation of the results with other authors. EH took part in interpreting the results and also participated in preparing the manuscript for submission. DSK contributed to interpreting the results and the discussions. All authors read and approved the final manuscript.

## Pre-publication history

The pre-publication history for this paper can be accessed here:

http://www.biomedcentral.com/1472-6963/14/100/prepub

## Supplementary Material

Additional file 1 **The exchange rate listed by The Bank of Korea.** (http://ecos.bok.or.kr/).
Click here for file

Additional file 2 Drug expenditure trends for the seven different statins (Korean currency, won). Click here for file
