# Supplementary material for: Effective policy initiatives to constrain lipid-lowering drug expenditure growth in South Korea
Source: BMC Health Serv Res. 2014 Mar 3;14:100. doi: 10.1186/1472-6963-14-100 (PMC4015215; doi:10.1186/1472-6963-14-100)
Supplement: Additional file 1 — The exchange rate listed by The Bank of Korea. (http://ecos.bok.or.kr/). [file 1472-6963-14-100-S1.docx]

The exchange rate listed by The Bank of Korea.( <http://ecos.bok.or.kr/> )

| Year | Exchange rate (W/US$) |
| --- | --- |
| 2001 | 1,290.8 |
| 2002 | 1,251.2 |
| 2003 | 1,191.9 |
| 2004 | 1,144.7 |
| 2005 | 1,024.3 |
| 2006 | 955.5 |
| 2007 | 929.2 |
| 2008 | 1,102.6 |
| 2009 | 1,276.4 |
| 2010 | 1,156.3 |
| 2011 | 1,108.1 |
