# Supplementary material for: Effective policy initiatives to constrain lipid-lowering drug expenditure growth in South Korea
Source: BMC Health Serv Res. 2014 Mar 3;14:100. doi: 10.1186/1472-6963-14-100 (PMC4015215; doi:10.1186/1472-6963-14-100)
Supplement: Additional file 2 — Drug expenditure trends for the seven different statins (Korean currency, won). [file 1472-6963-14-100-S2.docx]

Figure Drug expenditure trends for the seven different statins(Korean currency, won)
